# Revised Heart Allocation Policy Improved Waitlist Mortality and Waiting Time With Maintained Outcomes in En-Bloc Heart-Lung Transplant Candidates and Recipients

**DOI:** 10.3389/ti.2023.11956

**Published:** 2023-12-13

**Authors:** Yasuhiro Shudo, Hao He, Stefan Elde, Y. Joseph Woo

**Affiliations:** Stanford Healthcare, Stanford, CA, United States

**Keywords:** heart-lung transplant, heart allocation policy, waitlist mortality, post-transplant outcomes, waitlist outcomes

## Abstract

The revised United Network for Organ Sharing heart allocation policy was implemented in October 2018. Using a national transplant database, this study evaluated the transplant rate, waitlist mortality, waiting time, and other outcomes of en-bloc heart-lung transplantation recipients. Adult patients registered on the national database for heart-lung transplants before and after the policy update were selected as cohorts. Baseline characteristics, transplant rates, waitlist mortality, waiting times, and other outcomes were compared between the two periods. In total, 370 patients were registered for heart-lung transplants during the pre- and post-periods. There were significantly higher transplant rates, shorter waitlist times, and substantially reduced waitlist mortality in the post-period. Registered patients waitlisted in the post-period had significantly higher utilization of intra-aortic balloon pumps, extracorporeal membrane oxygenation, and overall life support, including ventricular assist devices. Transplant recipients had significantly longer ischemic times, increased transport distances, and shorter waiting times before transplantation in the post-policy period. Transplant recipients held similar short-term survival before and after the policy change (log-rank test, *p* = 0.4357). Therefore, the revised policy significantly improved access to en-bloc heart-lung allografts compared with the prior policy, with better waitlist outcomes and similar post-transplant outcomes.

## Graphical Abstract

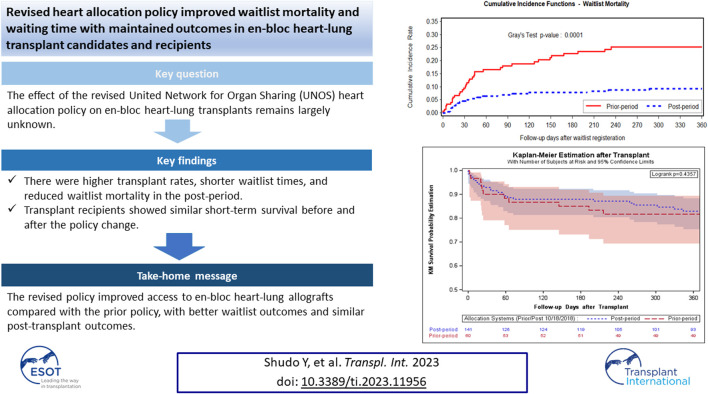

## Introduction

En-bloc heart-lung transplantation (HLTx) is well-established as an effective and definitive treatment for patients with advanced cardiopulmonary failure. Since the first successful operation performed in 1981 [1], >3,200 patients have undergone HLTx worldwide [2, 3].

Furthermore, to optimize the utilization of scarce donor hearts, the United Network for Organ Sharing (UNOS) revised the heart allocation policy in the United States, which took effect on 18 October 2018 [4]. Briefly, the new policy stratifies recipient candidates into six statuses and prioritizes transplantation of patients requiring temporary mechanical circulatory support, such as extracorporeal membrane oxygenation (ECMO) or an intra-aortic balloon pump (IABP) [4]. Previous studies have also assessed changes in post-transplantation outcomes associated with the allocation policy change. However, most of these studies examined isolated heart transplantation, and the effect of this change on multi-organ transplants remains largely unknown.

This study aimed to evaluate the transplantation rate, waitlist mortality, waiting time, and other outcomes of HLTx candidates and recipients using a national transplantation database.

## Materials and Methods

This study was based on National Organ Procurement and Transplantation Network STAR database (UNOS) released in January 2023. First-time HLTx registrants aged >18 years were selected from the UNOS database. Two periods were defined to compare the demographic characteristics and outcomes between the previous (pre-period) and new (post-period) allocation systems. Each period was 3.5 years, and the time of year was matched in both periods. The pre-period cohort was defined as patients who registered for HLTx between 18 October 2014, and 17 April 2018; similarly, the post-period was between 18 October 2018, and 17 April 2022. Thus, all patients who were listed within a designed period, but still waiting in waitlist by the end of this period, or died/transplanted/delisted after this period were treated as “censored” in these time-to-event-analyses.

The primary outcomes were waitlist mortality, defined as death from waitlist registration, and overall transplant mortality, defined as death from transplantation. Other waitlist outcomes, such as transplant rate and transplanted patients’ hospitalization outcomes, such as graft failure episodes, were assessed and compared between periods.

Continuous variables were described as means ± standard deviation or as medians with interquartile ranges (IQR) (25th and 75th percentiles) as appropriate. The continuous variables were compared using the Student’s t-test for mean differences and the Wilcoxon rank-sum test for median differences. Categorical variables were compared using the *χ* ^2^ test or Fisher’s exact test. Cumulative Incidence Functions (CIF) curves showed tendencies of waitlist mortalities in two periods of time. Gray’s cumulative risk test was used to test CIF curves when considering transplanted events and delisted events as competing risks. Kaplan-Meier (KM) survival curves were created to visually depict the overall survival of transplanted groups, and the log-rank test was used to test KM curves of two periods. The Cox proportional hazards models were used to estimate the unadjusted and adjusted hazard ratios of periods on three possible events (transplanted, dead while waiting and delisted) when patients were waiting on the waitlist. When one interested event was estimated, the other two were treated as competing risks. Adjusted hazard ratios were obtained after adjustments of multiple demographic and clinical factors, which included patient age when registering on the waitlist, gender, race, prior cardiac surgery, ECMO at the listing, IABP at the listing, Ventilator at the listing, VAD at the listing, Life Support at the listing, and Other Mechanism Life Support at the listing. For all statistical analyses, statistical significance was set at a two-sided level of 0.05. All analyses were performed using SAS version 9.4 (SAS Institute Inc., Cary, NC, USA). This study was based on Organ Procurement and Transplantation Network data as of 4 September 2020.

## Results

**FIGURE 1:**
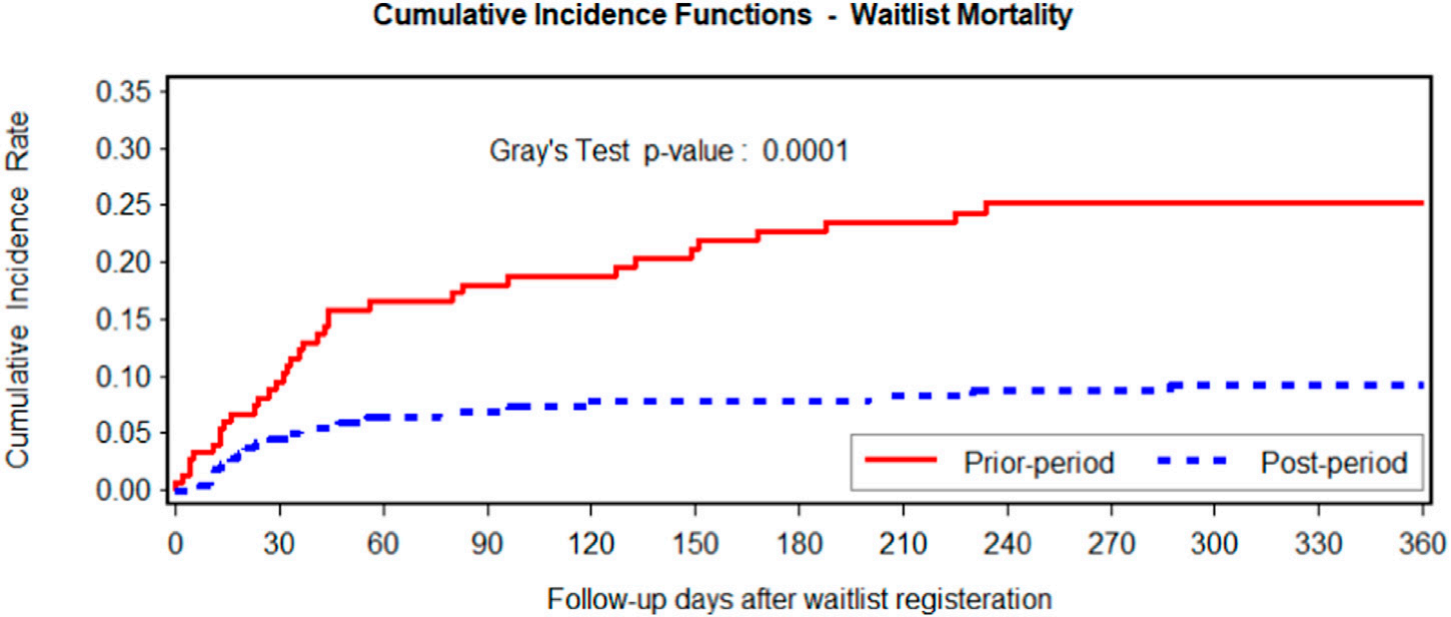
Fine-Gray cumulative incidence function comparing waitlist mortality using transplantation or delisting as competing events. The post-period is in blue, and the pre-period is in red. Fine-Gray *p*-value of .0001 on the cumulative incidence rates of the two groups indicates that waitlist mortality significantly decreased after the allocation change.

The demographic data and characteristics of the waitlist cohort are presented in Table 1. There were 152 patients listed who registered for heart-lung transplantation between 18 October 2014, and 17 April 2018, and 218 between 18 October 2018, and 17 April 2022. Of these patients, 60 recipients (39.5%) underwent transplantation pre-period and 141 (64.7%) post-period. There was a significantly higher transplantation rate (141/218 vs. 60/152, *p* < 0.001) in the post-period. There was no significant difference in age (*p* = 0.194), gender (*p* = 0.599); however, race (*p* = 0.118), blood type (*p* = .0105), and BMI (*p* = 0.415) differed significantly between periods. Patients registered for HLTx in the post-period had significantly higher utilization of IABP (4.1% vs. 0%, *p* = 0.012), ECMO (21.1% vs. 9.9%, *p* = 0.004), and overall life support including ventricular assist devices (VAD) (44.5% vs. 30.9%, *p* = 0.008) while waitlisted. Moreover, there was a significantly shorter waitlist time (164 ± 244 days vs. 253 ± 373 days, *p* = 0.009) in the post-period. The higher utilization of IABP, ECMO, and overall life support, including VAD, suggests that the cohort registered for HLTx in the current era included recipients with relatively more severe illnesses. Notably, however, the waitlist mortality in the post-period was significantly reduced (11.0% vs. 23.4%, competing risks Gray’s test *p* = 0.0001) (Figure 1).

**TABLE 1:** Demographic data of the waitlist cohort.

42 months Waitlist cohort N = 370		Pre-period (10/18/2014– 4/17/2018) *n* = 152	Post-period (10/18/2018– 4/17/2022) *n* = 218	*p*-value
Age (y)	Mean ± SD	44.0 ± 12.7	45.7 ± 12.4	0.194
	Median [IQR]	45.5 [33.5, 55]	47 [35, 56]	0.172
Gender	Female, n (%)	69 (45.4%)	105 (48.2%)	0.599
	Male, n (%)	83 (54.6%)	113 (51.8%)	
Race	White, n (%)	114 (75.0%)	147 (67.4%)	0.118
	Black, n (%)	26 (17.1%)	57 (26.2%)	
	Others, n (%)	12 (7.9%)	14 (6.4%)	
Blood Type	A, n (%)	37 (24.3%)	73 (33.5%)	0.105
	B, n (%)	25 (16.5%)	42 (19.3%)	
	O, n (%)	84 (55.3%)	99 (45.4%)	
	AB, n (%)	6 (4.0%)	4 (1.8%)	
BMI (kg/m^2^)	Mean ± SD	24.3 ± 4.8	24.8 ± 4.8	0.415
	Median [IQR]	24.0 [20.8, 28.0]	24.2 [21.0, 28.0]	0.538
Conditions at listing				
Prior Cardiac Surgery	n (%)	42 (27.6%)	63 (28.9%)	0.791
ECMO at listing	n (%)	15 (9.9%)	46 (21.1%)	0.004
IABP at listing	n (%)	0 (0.0%)	9 (4.1%)	0.012
Ventilator at listing	n (%)	12 (7.9%)	19 (8.7%)	0.779
VAD at listing	n (%)	5 (3.3%)	9 (4.1%)	0.677
Other Mechanism Life Support at listing	n (%)	17 (11.2%)	37 (17.0%)	0.121
Life Support at listing (including VAD)	n (%)	47 (30.9%)	97 (44.5%)	0.008
Status at end-of-period	Transplanted, n (%)	60 (39.5%)	141 (64.7%)	<.001
	Died-while-waiting, n (%)	37 (23.4%)	24 (11.0%)	
	Delisted, n (%)	16 (10.5%)	31 (14.2%)	
	Still waiting, n (%)	39 (25.7%)	22 (10.1%)	
Time on the waitlist (days)	Mean ± SD	253.3 ± 373.2	163.9 ± 243.7	0.009
	Median [IQR]	137.5 [29, 310.5]	54.5 [14, 230]	0.002

Categorical variables are expressed as n (%), and continuous variables are expressed as mean ± standard deviation or as median with interquartile range (IQR) (25th and 75th percentiles). BMI, body mass index; IABP, intra-aortic balloon pump; ECMO, extracorporeal membrane oxygenation; VAD, ventricular assist device.

As shown in Table 2, recipients in the transplanted cohort were, on average, 1.6 years older in the post-period than those in the pre-period, although not significant (44.4 ± 13.1 years vs. 46.0 ± 12.2 years; *p* = 0.405). It was very similar for donor age between the two periods (post-period, 32.9 ± 11.5 years vs. pre-period, 32.9 ± 12.7 years; *p* = 0.996). The distribution of transplants between sexes in each period was 53.2% and 58.3% in the post- and pre-periods, respectively (*p* = 0.503), and the proportion of recipient-to-donor sex matches decreased (71.6% vs. 76.7%, in the post- and pre-periods, respectively; *p* = 0.461). No significant difference was observed in recipient blood type (*p* = 0.661) or blood type match between the post- and pre-periods (84.4% and 90.0%, respectively; *p* = 0.376). The mean recipient body mass index was similar between eras (post-period, 24.4 ± 4.9 kg/m^2^, and pre-period, 23.7 ± 5.2 kg/m^2^; *p* = 0.331).

**TABLE 2:** Demographic data of transplanted cohort.

Transplanted groups in a 42 months Waitlist cohort N = 201		Pre-period (10/18/2014– 4/17/2018) *n* = 60	Post-period (10/18/2018–4/17/2022 *n* = 141	*p*-value
Prior Transplant Demographic Data				
Recipient age (y)	Mean ± SD	44.4 ± 13.1	46.0 ± 12.2	0.405
	Median [IQR]	46 [32.5, 55.5]	47 [38, 56]	0.468
Donor age (y)	Mean ± SD	32.9 ± 12.7	32.9 ± 11.5	0.996
	Median [IQR]	32 [22, 43.5]	33 [24, 41]	0.899
Gender	Female, n (%)	25 (41.7%)	66 (46.8%)	0.503
	Male, n (%)	35 (58.3%)	75 (53.2%)	
Gender Match (recipient to donor)	n (%)	46 (76.7%)	101 (71.6%)	0.461
Race	White, n (%)	47 (78.3%)	98 (69.5%)	0.173
	Black, n (%)	8 (13.3%)	35 (24.8%)	
	Others, n (%)	5 (8.3%)	8 (5.7%)	
Recipient Blood Type	A, n (%)	20 (33.3%)	59 (41.8%)	0.661
	B, n (%)	14 (23.3%)	26 (18.4%)	
	O, n (%)	24 (40.0%)	53 (37.6%)	
	AB, n (%)	2 (3.3%)	3 (2.1%)	
Blood Type Match	n (%)	54 (90.0%)	119 (84.4%)	0.376
(recipient to the donor)				
Recipient BMI (kg/m^2^)	Mean ± SD	23.7 ± 5.2	24.4 ± 4.9	0.331
	Median [IQR]	21.9 [20.0, 27.2]	24.1 [20.9, 27.5]	0.165
Indication for Transplant	Congenital heart disease, n (%)	16 (26.7%)	23 (16.3%)	0.301
	Pulmonary Hypertension, n (%)	16 (26.7%)	51 (36.2%)	
	Pulmonary fibrosis, n (%)	12 (20.0%)	32 (22.7%)	
	Other, n (%)	16 (26.7%)	35 (24.8%)	
Prior Cardiac Surgery	n (%)	23 (38.3%)	42 (30.0%)	0.236
Prior Lung Surgery	n (%)	1 (1.7%)	2 (1.4%)	0.994
Operative Data				
Ischemic Time (hrs)	Mean ± SD	3.6 ± 0.9	3.9 ± 0.9	0.006
	Median [IQR]	3.5 [3.1, 4.1]	4.0 [3.4, 4.5]	0.004
Distance, donor hospital to transplant center (miles)	Mean ± SD	129.4 ± 52.9	235.5 ± 201.4	<.001
	Median [IQR]	53 [12.5, 214]	207 [66, 380]	<.001
Time on the waitlist (days)	Mean ± SD	135.0 ± 140.5	96.0 ± 135.9	0.067
	Median [IQR]	117 [23.5, 191.5]	38 [9, 121]	0.008
Preoperative Life Support				
ECMO at listing	n (%)	6 (10.0%)	33 (23.4%)	0.032
ECMO at transplant	n (%)	12 (20.0%)	44 (31.2%)	0.123
IABP at listing	n (%)	0 (0.0%)	8 (5.7%)	0.108
IABP at transplant	n (%)	1 (1.7%)	13 (9.2%)	0.069
Ventilator at listing	n (%)	4 (6.7%)	14 (9.9%)	0.594
Ventilator at transplant	n (%)	7 (11.7%)	13 (9.2%)	0.612
VAD at listing	n (%)	1 (1.7%)	4 (2.8%)	0.981
VAD at transplant	n (%)	2 (3.3%)	4 (2.8%)	0.997
Other Mechanism Life Support at listing	n (%)	11 (18.3%)	28 (19.9%)	0.803
Other Mechanism Life Support at transplant	n (%)	16 (26.7%)	25 (17.7%)	0.151
Life Support at listing (including VAD)	n (%)	22 (36.7%)	68 (48.2%)	0.132
Life Support Pre-transplant (including VAD)	n (%)	30 (50.0%)	84 (59.6%)	0.211
Post Transplant Outcomes				
Length of stay (days)	Mean ± SD	52.7 ± 57.4	50.7 ± 54.0	0.815
	Median [IQR]	33 [21, 53]	36 [20, 57]	0.706
Stoke	n (%)	6 (10.0%)	8 (5.7%)	0.363
Dialysis	n (%)	20 (33.3%)	37 (26.2%)	0.307
PPM	n (%)	2 (3.3%)	2 (1.4%)	0.231
Airway	n (%)	0 (0.0%)	5 (3.6%)	0.469
Graft Failure	n (%)	24 (40.0%)	35 (24.8%)	0.031
In-hospital mortality	n (%)	7 (11.7%)	14 (9.9%)	0.802

Categorical variables are expressed as n (%), and continuous variables are expressed as mean ± standard deviation or as median with interquartile range (IQR) (25th and 75th percentiles). BMI, body mass index; IABP, intra-aortic balloon pump; ECMO, extracorporeal membrane oxygenation; VAD, ventricular assist device; PPM, permanent pacemaker.

**FIGURE 2:**
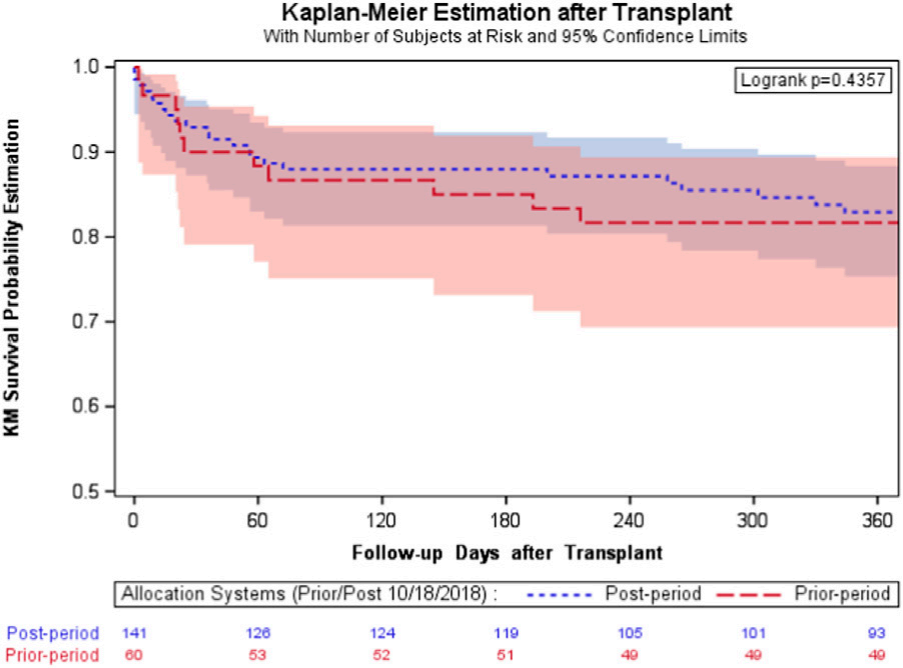
The survival curves after transplantation of the transplanted subgroup. Overall survival Kaplan-Meier estimates stratified between prior (red) and post (blue) policy change: a similar short-term survival before and after policy change (log-rank test, *p* = .4357).

Transplant recipients receiving HLTx within the post-period tended to have higher utilization of IABP (9.2% vs. 1.7%, *p* = 0.069) and ECMO (31.2% vs. 20.0%, *p* = 0.123) at transplant. However, the difference between periods was not significant. HLTx recipients also had significantly longer ischemic times (Medians 4.0 h vs. 3.5 h, *p* = 0.004) and shorter waiting times before transplantation (median 38 days vs. 117 days, *p* = 0.008) in the post-period following the policy change. Donor organs were transported from significantly farther distances in the post-period than in the pre-period, with the mean distance from the donor hospital to the recipient transplant center being 235.5 ± 201.4 miles in the post-period compared with 129.4 ± 52.9 miles in the pre-period (*p* < 0.001). There was no significant difference in distribution of indications for transplantation (*p* = 0.301). Fewer patients in the post-period had a history of prior cardiac surgery than those in the pre-period (30.0% vs. 38.3%, *p* = 0.236); however, the difference was insignificant. The median length of hospital stay during transplant hospitalization was similar between periods (post-period, 36 days; interquartile ranges (IQR), 20–57 days and pre-period, 33 days; interquartile ranges (IQR), 21–53 days; *p* = 0.706). There were higher risks; however, transplant recipients showed a significantly lower graft failure rate (24.8% vs. 40.0%, *p* = 0.031). Further, these patients tended to have a lower in-hospital mortality (9.9% vs. 11.7%, *p* = 0.802), thus having similar short-term survival before and after the policy change (log-rank test, *p* = 0.4357) (Figure 2).

Conversely, 24 listed patients (11.0%) died waiting for a transplant in the new allocation system, whereas 37 recipients (24.3%) died in the pre-period. The waitlist mortality rate was significantly lower (24/218 vs. 37/152, *p* < 0.001) during the post-period. As shown in Table 3, recipients in the waitlist mortality cohort were, on average, 4.7 years older in the post-period than those in the pre-period (44.6 ± 12.3 years vs. 50.3 ± 11.3 years; *p* = 0.079). No significant differences in sex (*p* = 0.903) or body mass index (*p* = 0.539) were observed between the two periods. Recipients who died while waiting for transplantation within the post-period showed a significantly higher utilization of life support (70.8% vs. 35.1%, *p* = 0.009) while waitlisted.

**TABLE 3:** Demographic data of patients who died while waiting for transplant.

Died-while-waiting groups in 42 months Waitlist cohort N = 61		Pre-period (10/18/2014– 4/17/2018) *n* = 37	Post-period (10/18/2018– 4/17/2022) *n* = 24	*p*-value
Age (y)	Mean ± SD	44.6 ± 12.3	50.3 ± 11.3	0.079
	Median [IQR]	47 [37, 55]	53.5 [42, 60]	0.077
Gender	Female, n (%)	16 (43.2%)	10 (41.7%)	0.903
	Male, n (%)	21 (56.8%)	14 (58.3%)	
Race	White, n (%)	30 (81.1%)	16 (66.7%)	0.050
	Black, n (%)	4 (10.8%)	8 (33.3%)	
	Others, n (%)	3 (8.1%)	0 (0.0%)	
BMI (kg/m^2^)	Mean ± SD	24.3 ± 4.3	25.0 ± 4.9	0.539
	Median [IQR]	24.0 [22.0, 26.6]	24.2 [21.4, 28.1]	0.854
Conditions at listing				
Prior Cardiac Surgery	n (%)	7 (18.9%)	7 (29.2%)	0.353
ECMO at listing	n (%)	5 (13.5%)	7 (29.2%)	0.189
IABP at listing	n (%)	0 (0.0%)	1 (4.2%)	0.393
Ventilator at listing	n (%)	6 (16.2%)	3 (12.5%)	0.776
VAD at listing	n (%)	2 (5.4%)	4 (16.7%)	0.201
Other Mechanism Life Support at listing	n (%)	3 (8.1%)	6 (25.0%)	0.136
Life Support at listing (including VAD)	n (%)	13 (35.1%)	17 (70.8%)	0.009
Time on the waitlist (days)	Mean ± SD	85.8 ± 117.5	144.5 ± 192.9	0.189
	Median [IQR]	37 [16, 127]	41.5 [17, 215]	0.562

Categorical variables are expressed as n (%), and continuous variables are expressed as mean ± standard deviation or as median with interquartile range (IQR) (25th and 75th percentiles). BMI, body mass index; IABP, intra-aortic balloon pump; ECMO, extracorporeal membrane oxygenation; VAD, ventricular assist device; PPM, permanent pacemaker.

Considering the competing risks of waitlist outcomes and controlling for possible confounding factors, the Cox Proportional Hazards regression models were used to estimate the periods’ unadjusted and adjusted hazard ratios (Table 4). Notably, the unadjusted and adjusted hazard ratios for transplants within periods and death while waiting for transplants were statistically significant. In particular, the hazard ratios of pre-period vs. post-period of transplants within the periods were 0.511 without covariate adjustments and 0.544 after adjustments of covariates (*p* < 0.001 for both), indicating that the transplant likelihood during pre-period was around half of that during post-period. Conversely, the hazard ratios of pre-period vs. post-period of death while waiting for transplants were 2.609 without covariate adjustments and 2.852 after adjustments (*p* < 0.001 for both), which indicated that the death likelihood while waiting for HLTx during pre-period was over 2.6 times of the death likelihood during post-period. This is strong evidence that the new allocation policy has significantly improved patients’ survival and saved lives. Notably, there were no significant differences in delisting within these periods.

**TABLE 4:** Hazard ratios of pre-period vs. post-period from Cox PH models.

Specific Event of Interest	# of Interest Event	# of Competing Events	# of Censored	Hazard Ratio Type	HR of Interest Event: Pre-period vs. Post-period	95% CI of HR	P-value
Died While Waiting							
	61	248	61	Unadjusted	2.609	[1.564, 4.351]	<.001
				Adjusted^a^	2.852	[1.670, 4.868]	<.001
Transplanted Within Periods							
	201	108	61	Unadjusted	0.511	[0.381, 0.686]	<.001
				Adjusted^a^	0.544	[0.401, 0.736]	<.001
Delisted Within Periods							
	47	262	61	Unadjusted	0.794	[0.436, 1.445]	0.451
				Adjusted^a^	0.789	[0.413, 1.507]	0.473

HR, Hazard ratio; CI, confidence interval.

^a^ Results adjusted in Cox proportional hazards model by baseline characteristics—patient age when registering on the waitlist, gender, race, prior cardiac surgery, ECMO at the listing, IABP at the listing, ventilation at the listing, VAD at the listing, Life Support at the listing, and other mechanical life support at the listing.

## Discussion

This comprehensive study investigated the impact of the revised UNOS heart allocation policy on transplant rate, waitlist mortality, waiting time, and other outcomes of adult primary HLTx recipients using the UNOS STAR database. We stratified the cohort by disjoint categories of patients registered for HLTx in the allocation system during the previous period, before the policy update (10/2015–04/2018), as well as during the period post-policy update (10/2018–04/2021).

The UNOS updated its heart allocation policy in the United States in October 2018 [4]. Notably, more categories were introduced to better stratify the urgency for recipients of heart transplants, from three categories (status 1A, 1B, and 2) to six categories (status 1–6). These changes were fundamentally implemented to decrease mortality rates for recipients on the waiting list, while providing an opportunity for others to receive organs.

Regarding the allocation of heart and lung combinations, when heart-lung transplantation candidates are registered on the heart, lung, and heart-lung waiting lists, the second organ is allocated to the heart-lung transplantation candidate from the same donor. In practice, donor organs are allocated by running a list of hearts for each recipient. In reality, if the heart offer comes as a primary offer to the heart-lung transplantation candidate, lungs must be offered from the same donor, even if the heart-lung transplantation candidate’s need for those lungs is far less urgent than for others on the lung list.

A potential concern was that the new organ allocation system might lead to disadvantages for heart-lung transplantation recipients, since heart-lung transplantation candidates are generally listed as status 4 or 5 in the new system. However, they were listed as status 1B or 2 in the previous system unless they had higher requirements for ECMO, IABP, or other mechanical life support, prolonging the waiting period [5]. Nevertheless, our data showed that the cohort from the new allocation system was associated with higher transplant rate, reduced waitlist mortality, and shorter waiting time. Based on this analysis, the revised heart allocation policy significantly improves access to en-bloc heart-lung allografts than the prior policy, with better waitlist outcomes.

Our data also showed that recipients who underwent transplantation during the new allocation system included baseline demographics indicating more severe illness, as evidenced by higher utilization of IABP, ECMO, and overall life support, including VAD at transplantation. One may argue that maintaining patients on ECMO in the preoperative phase has been reported as a high-risk resource [3], yet it seems that it has become commonplace in many of our institutions. This study found that >30% of heart-lung transplant recipients were on ECMO at the time of transplant. Nevertheless, the equivalent graft survival was demonstrated by short-term mortality in our study. This result is supported by our institution’s previous report, which focused on reasonable outcomes among adult transplant recipients who underwent HLTx bridged from ECMO [6].

In addition to the transplanted recipients’ demographics that have been mentioned earlier, the characteristics of patients registered for transplantation who died while waiting were equally important in this study. Our data showed that >70% of patients in the waitlist mortality cohort were on life support, including VAD. This could likely be explained by insufficient access to organs for HLTx candidates with severe illness, and this issue should be addressed in future studies.

Finally, we appear to have made good progress in the pre-transplant phase, with decreased waitlist mortality and faster time to transplant for patients requiring heart-lung transplants. Conversely, post-transplant outcomes seem to have plateaued across the eras. This issue may be partly resolved with the newly developed innovative organ preservation and transport system, which may positively impact long-term survival in this complex patient population [7].

### Limitations of the Database

This study has limitations consistent with those of retrospective analyses and the use of a national multicenter database. The UNOS database has some considerable uncollected data for crucial factors during specific periods; however, the UNOS/OPTN registry provided a large sample size to assess the influence of the revised UNOS heart allocation policy on the transplant rate, waitlist mortality, waiting time, and other outcomes of adult HLTx recipients. However, specific recipient characteristics may also contribute to recipient mortality; several have not been included in our analysis. A potential selection bias may have existed wherein physicians believe that obesity is a prohibitive risk factor for HLTx. In addition, only donors whose organs were accepted for transplantation were included. The selection of a suitable donor is a complicated process. Clinicians must consider multiple factors, evaluating recipient urgency against donor characteristics, ischemic time, recipient sensitization, and donor/recipient size mismatches. Therefore, additional characteristics may be responsible for post-transplant graft failure, and these factors were not considered in this analysis.

### Conclusion

The revised UNOS policy was associated with higher transplant rates, reduced waitlist mortality, shorter waiting times, and similar post-transplant short-term survival rates. Based on this analysis, the revised heart allocation policy significantly improved access to en-bloc heart-lung allografts than the prior policy, with better waitlist outcomes and similar post-transplant outcomes.

## Data Availability

The original contributions presented in the study are included in the article/supplementary material, further inquiries can be directed to the corresponding author.

## Abbreviations

ECMO, extracorporeal membrane oxygenation; HLTx, heart-lung transplantation; IABP, intra-aortic balloon pump; UNOS, United Network for Organ Sharing; VAD, ventricular assist device.
